# Combined Biomaterials: Amniotic Membrane and Adipose Tissue to Restore Injured Bone as Promoter of Calcification in Bone Regeneration: Preclinical Model

**DOI:** 10.1007/s00223-020-00793-1

**Published:** 2021-01-09

**Authors:** Dilcele Silva Moreira Dziedzic, Júlio César Francisco, Bassam Felipe Mogharbel, Ana Carolina Irioda, Priscila Elias Ferreira Stricker, Juliana Floriano, Lúcia de Noronha, Eltyeb Abdelwahid, Célia Regina Cavichiolo Franco, Katherine Athayde Teixeira de Carvalho

**Affiliations:** 1 Cell Therapy and Biotechnology in Regenerative Medicine Department, The Pelé Pequeno Príncipe Institute, Child and Adolescent Health Research & Pequeno Príncipe Faculties, Ave. Silva Jardim, no. 1632, Box 80240-020, Curitiba, Paraná Brazil; 2 Positivo University, St.Professor Pedro Viriato Parigot de Souza, Box 80710-570, Curitiba, Paraná 5300 Brazil; 3 Physics Department, São Paulo State University (UNESP), Ave. Eng. Luís Edmundo Carrijo Coube, 2085 - Núcleo Res. Pres. Geisel, Box 17033-360, Bauru, São Paulo Brazil; 4 Pathology Department, The Institute of Biological and Health Sciences of the Pontifical Catholic University, Ave. Imaculada Conceição, 1155, Box 80215-901, Curitiba, Brazil; 5 Feinberg School of Medicine, Feinberg Cardiovascular Research Institute, Northwestern University, 303 E. Chicago Ave., Tarry 14–725, Chicago, IL 60611 USA; 6 Cell Biology Department, Federal University of Paraná, Ave. Coronel Francisco Heráclito dos Santos 210, Box 81531-970, Curitiba, Paraná Brazil

**Keywords:** Bone tissue engineering, Stem cell transplantation, Adipose stem cells, Human amniotic membrane

## Abstract

Discarded tissues, like human amniotic membranes and adipose tissue, were investigated for the application of Decellularized Human Amniotic Membrane (DAM) as a viable scaffold for transplantation of Adipose-derived stromal cells (ASCs) in bone regeneration of non-healing calvarial defects in rats. Amniotic membrane was decellularized to provide a scaffold for male Wistar rats ASCs expansion and transplantation. ASCs osteoinduction in vitro promoted the deposition of a mineralized bone-like matrix by ASCs, as calcified globular accretions associated with the cells on the DAM surface and inside the collagenous matrix. Non-healing calvarial defects on male Wistar rats were randomly divided in control without treatment, treatment with four layers of DAM, or four layers of DAM associated with ASCs. After 12 weeks, tissue blocks were examined by micro-computed tomography and histology. DAM promoted osteoconduction by increasing the collagenous matrix on both DAM treatments. DAM with ASCs stimulated bone deposition, demonstrated by a higher percentage of bone volume and trabecular bone number, compared to control. Besides the osteogenic capacity in vitro, ASCs stimulated the healing of calvarial defects with significant DAM graft incorporation concomitant with higher host bone deposition. The enhanced in vivo bone regeneration by undifferentiated ASCs loaded onto DAM confirmed the potential of an easily collected autologous cell source associated with a broadly available collagenous matrix in tissue engineering.

## Introduction

Bone presents continuous remodeling and a substantial regenerative capacity compared to other tissues. Lifelong bone remodeling is responsible for skeletal development, responses to mechanical stimuli, and maintaining mineral homeostasis. Intrinsic regeneration capacity guarantees bone integrity as an injury repair process. Moderate-sized bone defects repair without the need for a graft. However, complex clinical conditions require a considerable amount of bone, in which natural bone regeneration capacity is not sufficient to establish functional tissue recovery. Bone engineering strategies may be necessary to guide or accelerate the process, improving bone quantity or quality limitations, and as alternative methods to autologous bone grafts. Osteoconduction is the process of perivascular tissue precursor, and osteoprogenitor cell ingrowth, from the bony bed into implanted frameworks. Osteoinduction is the induction of undifferentiated mesenchymal stem cells into osteoprogenitor cells, also at ectopic sites. The combination of three-dimensional biocompatible frameworks with cells and growth factors may stimulate osteogenesis and osteoinduction, enhance the osteogenic capacity of transplanted and endogenous cells and thus, ensure better healing [1].

Commercially available collagen membranes are widely used in in vivo studies, associated with ceramic implant material [2], demineralized bone matrix [3], and growth factors [4]. The association of ceramic and membrane with Bone marrow mesenchymal stem cells (BMSCs) demonstrated to favor earlier bone deposition [5]. Tissues available in maternity hospitals, and often discarded, can be a reliable source of allogenous cells and collagenous scaffold [6], and the removal of the epithelial cell layer minimizes the risks of adverse immunological responses [7]. Amniotic membrane association with BMSCs [8] demonstrated that Decellularized Human Amniotic Membrane (DAM) is indicated as cell-carrier in tissue regeneration applications.

Studies using multipotent ASCs [9] have demonstrated their potential as a significant source of adult stem cells in regenerative medicine. Some significant advantages of ASCs in bone engineering compared to BMSCs are the facility in harvesting, higher cellular yield, and proliferation capacity [10]. The application of the patient autologous cells from fat, transferred in order to enrich and accelerate the bone regeneration process was reported, in cranioplasties associated with TCP [11], and graft [12]. Investigations on ASCs participation in calvarial bone defect repair have reported the occurrence of significant cell migration to the lesion site after intravenous cell administration [13], and paracrine effect of ASCs on in vitro and in vivo osteoblastic cell differentiation [14]. There was a significantly higher stimulation in cell association of immediately prepared defects, compared to cell graft with established bone defects [15]. There is considerable evidence indicating that ASCs cells may contribute also to periodontal regeneration [16]. The application of allogeneic decellularized amniotic membranes offers minimal possibility of rejection, for being an immune-privileged tissue [17] in the absence of the allogeneic amniotic epithelium [7, 18]. Findings of this study can be translated into clinics as an autologous tissue engineering treatment, by the association of a decellularized amniotic membrane, from a tissue bank or commercially available, with cells from an autologous source.

## Materials and Methods

The experimental design consisted primarily of the decellularization of a human amniotic membrane, expansion of adipose tissue-derived stromal cells from rats, and preparation of scaffolds for the treatment of non-healing calvarial defects of rats.

### Decellularized Human Amniotic Membrane

The human placenta was collected in accordance with The Code of Ethics of the World Medical Association, only after approval of the Human Research Ethics Committee (CEP) of Complexo Hospitalar Pequeno Príncipe and co-participating institution (Curitiba, Brazil; Number 659.204, 09/03/2015). The informed consent form was obtained from a mother undergoing natural childbirth at the Hospital Maternidade Victor Ferreira do Amaral (Curitiba, Brazil), after clinical screening for diseases. All experiments with animals complied with the ARRIVE guidelines, the national guidelines for the care and use of laboratory animals, and followed the protocols approved by the Animal Care Ethics Committee (CEUA) of Pequeno Príncipe Faculties (Curitiba, Brazil; Number 039-2018, 22/11/2018).

The amniotic membrane, collected immediately after placental expulsion in the surgical environment, was separated from the chorion, and handled within a laminar flow Class II Biosafe. The membrane was washed with Phosphate buffer saline (PBS 2% streptomycin/penicillin), trimmed into approximately 15 × 15 cm^2^ parts, kept in a decellularization solution (0.1% sodium dodecyl sulfate, SDS) on a horizontal mechanical shaker (120 rpm), followed by gentle scraping. Membranes were washed five times with PBS, cut into 8 mm in diameter circles with a surgical punch, stretched on culture dishes, kept with PBS, and exposed to UV light for 1 h inside the laminar flow. Before cell cultivation, DAM disks were incubated in complete regular cell culture medium (Dulbecco’s modified Eagle’s medium-F12, supplemented with 10% fetal bovine serum and 1% streptomycin/penicillin) in standard culture conditions of 5% CO_2_ in air at 37 °C for 72 h. Membrane decellularization and cell cultivation onto DAM disks were also observed after histological preparation of cross-sections: fixation with 10% natural buffered formalin, dehydration through increasing series of graduated ethanol, embedding in paraffin wax, sectioning at 4 µm thickness, and staining with by Hematoxylin and Eosin (H&E).

### Adipose-Derived Stromal Cells

Adipose-derived stromal cells were collected from the inguinal fat of eight-week-old male Wistar rats, after the animals were anesthetized by intraperitoneal administration 50 mg/Kg ketamine and 6.6 mg/Kg xylazine), and later euthanized through intracardiac administration of Thiopental 75 mg/kg. The Stromal vascular fraction (SVF) was obtained by collagenase digestion, according to previously published methods [9]. Shortly, adipose tissue was washed with PBS, macerated with two surgical blades, digested for 30 min at 37 °C in PBS containing 0.075% Type I collagenase. Digested tissue was centrifuged at 1200 rpm for 10 min after adding regular medium. Cells were suspended in PBS, passed in a 100 μm strainer, and centrifuged before suspension in regular medium. Nucleated cell yield was verified in a hemocytometer after Trypan Blue staining, and the initial plating density was 1 × 10^5^ cells/cm^2^ in T25 culture flasks. Cell culture flasks were incubated, non-adherent cells were removed after 72 h, and adherent cells were maintained with medium change every three days. Upon reaching 80% confluence, ASCs were transferred to another flask/dish after incubation with 0.25% trypsin/0.1% EDTA, expanded from a plating density of 1 × 10^3^ cells/cm^2^ in T75 culture flask in the second passage, and cryopreserved in 80% FBS, 10% medium, 10% dimethyl sulfoxide (DMSO). Cells were cultivated onto polystyrene dishes and on DAM at a density of 1.5 × 10^4^ cells/cm^2^ for proliferation.

### Cell Differentiation

Two different osteoinduction media were used separately after cell confluence, prepared with reagents known to favor the expression of the osteoblastic phenotype (50 mcg/mL Ascorbic acid, 10 mM β-Glycerophosphate, and 100 nM dexamethasone) diluted in regular medium, or commercially available Rat Osteoblast Differentiation Medium (Cell Applications, Inc; San Diego, USA). Either medium was added after cell confluence and changed every 3 days, up to 4 weeks of culture. Mineralization was observed by histochemical staining with Alizarin red staining (affinity for calcium), after fixation in 2.5% glutaraldehyde, washing 3 times with 70% ethanol, staining with 1% Alizarin red solution pH 5.5 for 5 min, washing 3 times with 50% ethanol, air-drying, and examination under a phase contrast microscope (Zeiss Axio Vert.A1, Zeiss; Jena, Germany) equipped with an AxioCam MRC camera (Zeiss, Germany). Membranes maintained with cells and regular medium or without cells in commercial osteoinduction medium for 4 weeks were used as controls. Observations in scanning electron microscopy (SEM) and X-ray energy dispersive spectroscopy (EDS) were performed on a TESCAN VEGA3 device (Kohoutovice, Czech Republic), in order to view and identify the mineral deposits in vitro, after sample fixation with Karnovsky, dehydration, critical point drying (for EDS), and gold metallization (for SEM). The average Ca/P atomic ratio was verified in specific demarcated points on Backscattered electron images (BSEI), based on elemental peak intensities after background subtraction and spectrum fitting.

Chondrogenic Differentiation Medium (Gibco; Carlsbad, USA) was used after the culture of cell micromass [19] (cell density of 1.6 × 10^7^ cells/ml) in quadruplicate in a 12-well plate. The adipogenic differentiation used medium prepared at the time of medium exchange with the addition of 0.5 μM dexamethasone, 0.5 mM of isobutylmethylxanthine, and 50 μM of indomethacin.

### Surgical Procedure and Harvesting

Before animal transplantation, ASCs were cultivated over the DAM disks (2.5 × 10^4^ cells/cm^2^) stretched onto 12-well plates. After cell confluence was observed on the polystyrene area around the membrane disks, in approximately 5 days, more cells were associated with the disks (2.5 × 10^4^ cells/cm^2^), 48 h before the surgical procedure. Fifteen Male Wistar rats (8 weeks old, weight 370 g) were randomly divided into three treatment groups: no DAM scaffold (T0 = control), DAM scaffold only (T1), and DAM scaffold associated with ASCs (T2).

The animals were anesthetized as described before. The hair over the cranial bones was shaved, the skin was aseptically prepared, and the animal head was stabilized in a stereotaxic frame (Stoelting Co.; Wood Dale, USA). A sagittal incision was made with a surgical blade through the skin over the calvaria, subcutaneous tissues were divulged, and the area was maintained exposed. The anatomic landmarks were identified, and the periosteum was incised and elevated with a blunt spatula. A single bicortical full-thickness defect was prepared on the midline using a low-speed dental surgical drilling unit (NSK 20:1 SMax SG20, Tochigi, Japan; Beltec LB 100, Araraquara, Brazil), with an 8 mm diameter trephine (Neodent; Curitiba, Brazil). Care was taken to avoid injury to the dura mater, with intermittent movement, and constant irrigation with saline solution. After the calvarial bone disk removal, the area was cleaned with abundant irrigation before the treatment. Four layers of DAM (T1) or four layers of DAM with ASCs (T2) were placed stretched one over one another on the intact dura mater at the defect base, without extending beyond the defect margins. The membranes were maintained in place with the overlying periosteum, and the surgical site was closed using a 7.0 polypropylene suture. Analgesic was administered for three days following the surgery. Euthanasia of the animals with the same procedure described, after 12 weeks of bone repair, was followed by specimen excision with a diamond disk, and one week of fixation with 10% natural buffered formalin.

### Tissue Processing and Imaging

Specimens were scanned with a SkyScan1174v2 Micro-computed tomography (micro-CT) scanner (50 Kv, 800 µA, pixel size 16.82 µm; Bruker micro-CT, Kontich, Belgium). Micro-CT images were reconstructed, aligned, visualized, and measured (Softwares NRecon, DataViewer, CTVox, and CTAn; Bruker micro-CT, Kontich, Belgium). For the measurement of various parameters with CTAn, a cylindrical ROI (9.3 mm in diameter) was placed on the center of the defect on a 2D image, enclosing all the new bone within the defect, and the defect margin. Measurement was done on 30 layers comprising the tridimensional defect volume after demarcating a threshold value from the gray level histogram. The following basic parameters were measured: Bone volume, Percentage of new bone volume in the total tissue volume (BV/TV), and Trabecular number (1/mm).

The specimens were decalcified with 10% EDTA (Ethylenediaminetetraacetic acid solution, pH 7.4) for three weeks, embedded in paraffin, and 4 µm histological sections were prepared, perpendicular to the sagittal suture. Deparaffinized sections were stained using H&E for baseline observation, and Picrosirius red stain (PRS), observed under linearly polarized light (POL). The PSR-POL method was used for morphometric image analysis of fifteen consecutive fields from histological sections of three specimens of each group. The collagenous content on the defect thickness was measured, including the periosteal and the meningeal sides, with the software Image-Pro Plus 7.0 (Media Cybernetics, Inc., Rockville, USA), quantifying the total polarized fiber content against the dark background. Statistical analysis was performed after the Shapiro–Wilk test for normality, using ANOVA along with post hoc Tukey’s test, or with non-parametric test Kruskal–Wallis and Kolmogorov–Smirnov test (STATISTICA software, Version 10, StatSoft Inc., USA). Results were presented in the text as the Mean ± Standard Error (SE), where *p < 0.05 was considered significant.

## Results

**Fig. 1:**
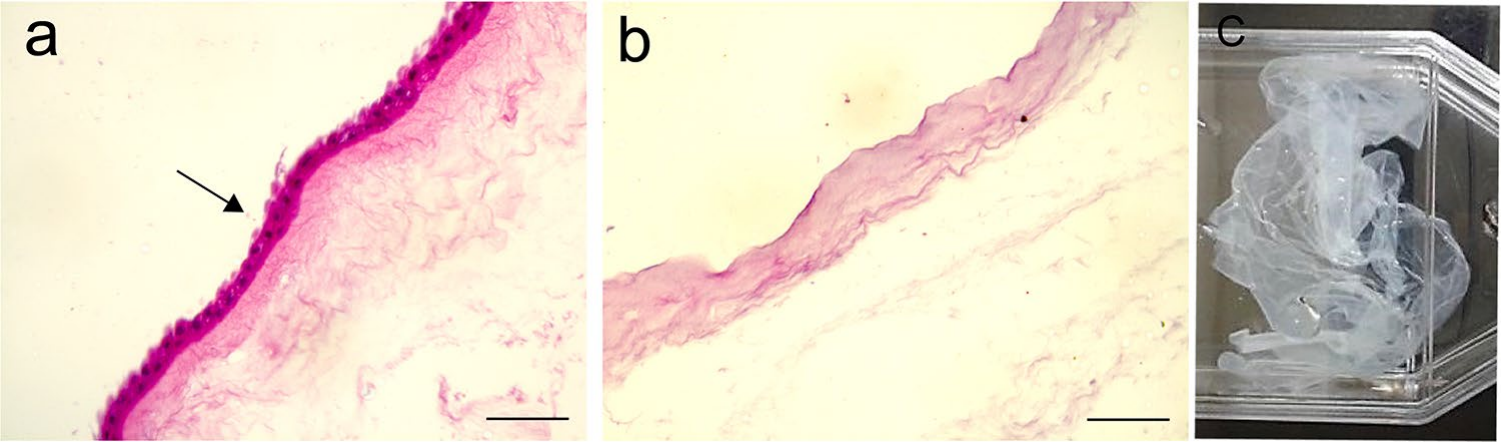
Human amniotic membrane. Histological cross-sections of the fresh and intact amniotic membrane before decellularization presented the epithelial cell layer (arrow, **a**) with purple nuclei, removed during the process which preserved the collagenous matrix (**b**). H&E stain, Objective × 20, Scale bars 100 µm. Decellularized membrane inside a T75 Cell Culture Flask (**c**)

The decellularization process with 0.1% SDS was effective in removing the cells and the innermost epithelial layer, which lies adjacent to the fetus (Fig. 1a). The structure of the subjacent collagenous layer was preserved (Fig. 1b), as a translucent collagenous acellular framework (Fig. 1c). The DAM preserved its structural integrity during the decellularization, and throughout the cell culture with regular medium, and both osteoinduction media.

**Fig. 2:**
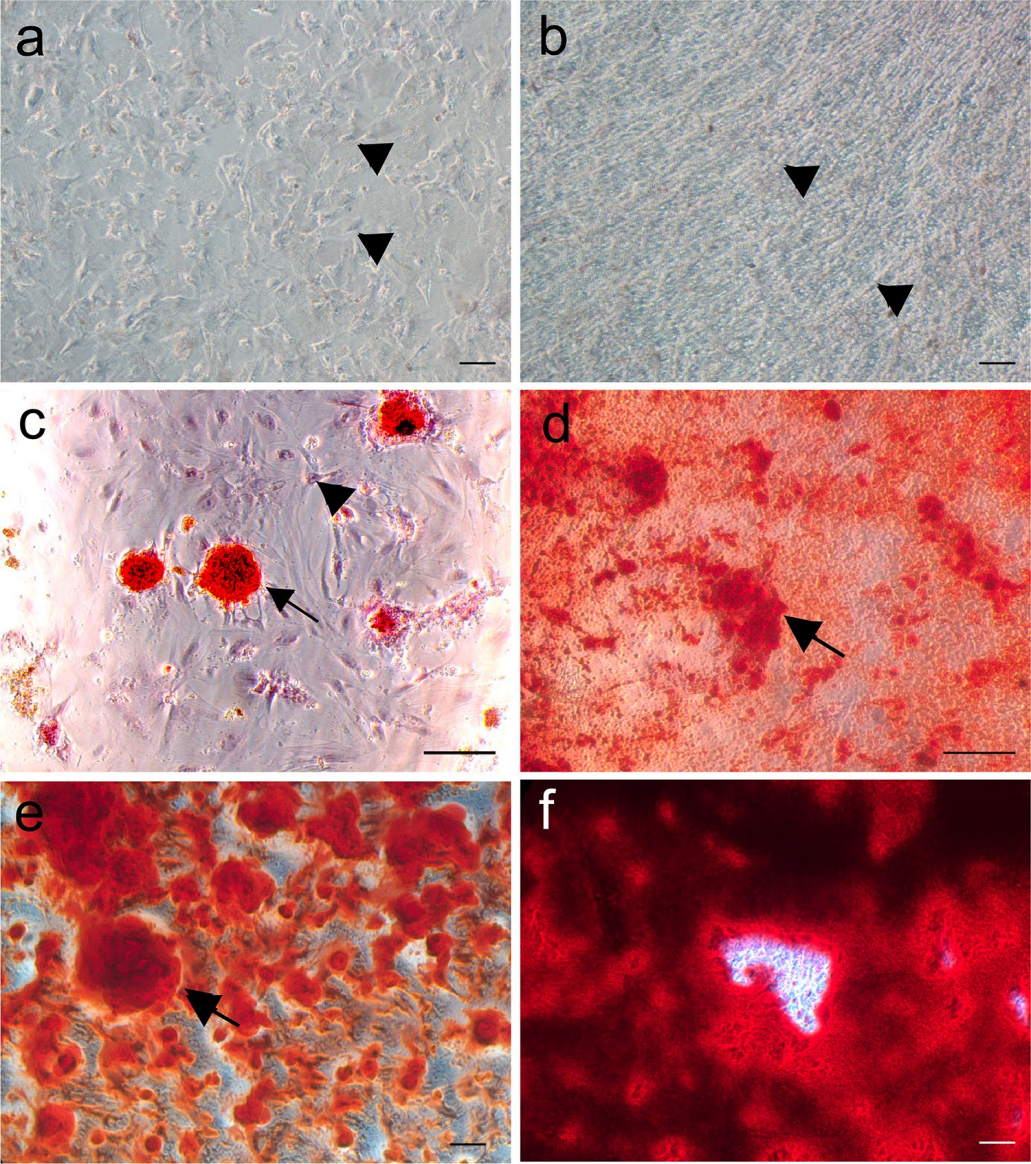
Representative areas of ASCs cultures for four weeks demonstrated osteogenic differentiation. Cultures with regular medium on polystyrene (**a**) and membrane (**b**) present cells (arrowhead) distributed on the dish and the membrane, without mineral deposits. Both osteoinduction media, prepared (**c**, **d**), and commercial (**e**, **f**), stimulated the deposition of a mineralized bone-like matrix, (arrow, red) on polystyrene (**c**, **e**) and the membrane (**d**, **f**). Alizarin Red stain; Objectives × 10 (**a**, **b**, **e**, **f**), × 20 (**c**, **d**); Scale bars 100 µm

**Fig. 3:**
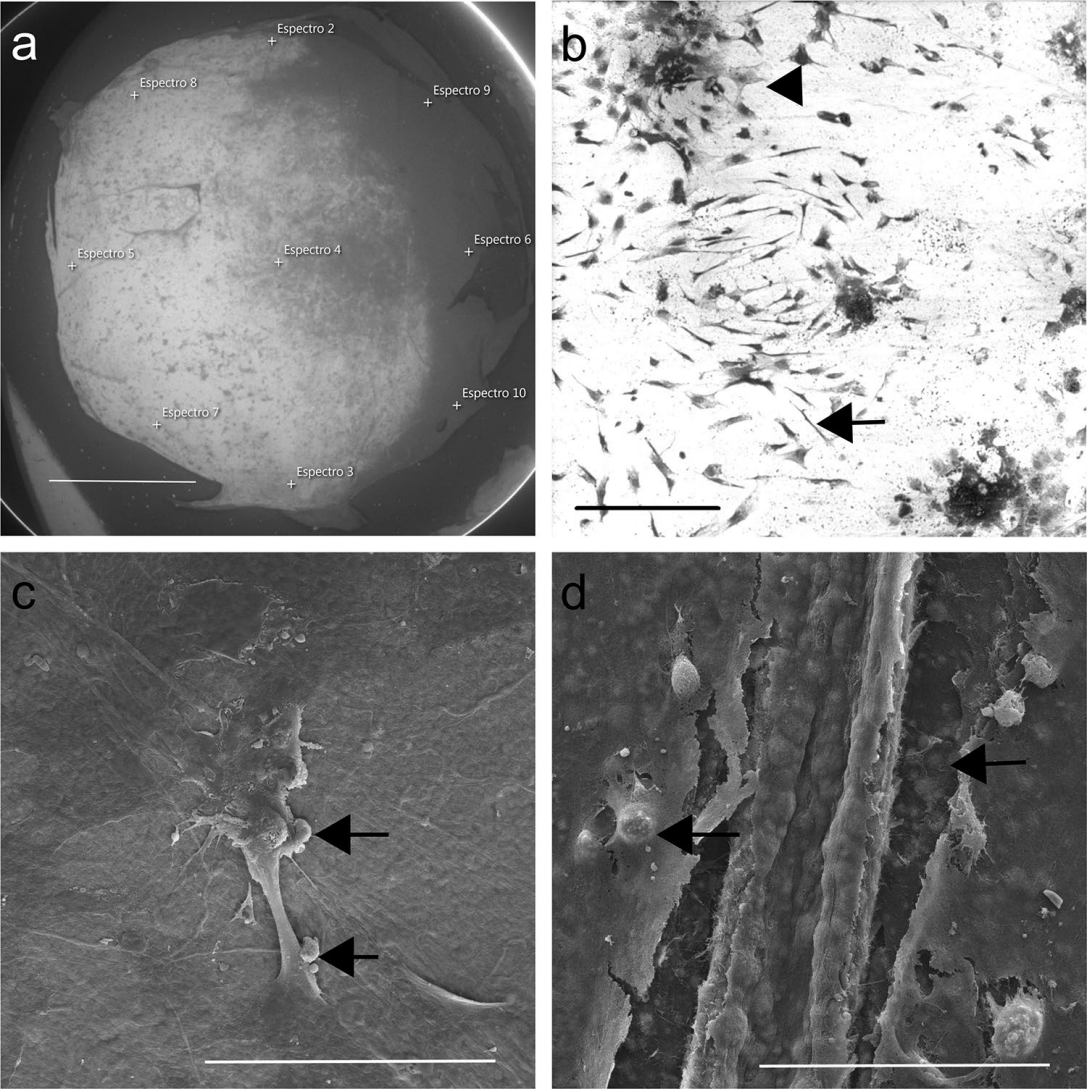
BSEI (**a**, **b**), and SEM observations (**c**, **d**). Large brighter area on membrane center and left side indicate higher atomic number, more mineralization (**a**, with demarcated points from EDS). Besides the Fibroblastic-like cell shape (arrow), ASCs also acquired a polygonal cell shape (arrowhead) on DAM surface (**b**). Extracellular matrix viewed as calcified globular accretions (arrow) associated with ASCs on membrane surface (**c**) and inside the collagenous scaffold (**d**). Areas with membrane tear resulted from artifact during membrane removal from culture dish after fixation (**a**) and critical point drying (**d**); Scale bars 2 mm (**a**), 200 µm (**b**), 50 µm (**c**, **d**)

ASCs with a density of 1.5 × 10^4^ cells/cm^2^ adhered and proliferated in culture on both substrates and reached confluence in about four days. Cell distribution on polystyrene dishes was easier to distinguish than on DAM (Fig. 2a). Cells on the DAM cultivated with regular medium remained most on the surface of the membrane and kept a more elongated, fibroblastic-like shape (Fig. 2b). The osteoinduction of ASCs on polystyrene and DAM promoted cell shape alteration to a more polygonal shape, associated with the gradual deposition of the Bone-like extracellular matrix (Fig. 2c–f). Both osteoinduction media stimulated the deposition of mineralized globular accretions on both substrates, first observed after 2 weeks, which increased in number and size, also coalescing (Fig. 2c–f). Mineralized matrix deposition by the ASCs associated with the DAM was more evenly distributed throughout the DAM (Figs. 2d, f, 3a), but on polystyrene was dispersed, in a patchy configuration (Figs. 2c, e).

Ultrastructural observations and mineral phase investigation by EDS elemental analysis (weight percentage), on DAM with ASCs and commercial osteoinductive medium for 4 weeks, allowed the observation of brighter areas coincident with more mineral deposition (Fig. 3a), and cell distribution onto the membrane (Fig. 3b).

Calcified globular accretions were observed beneath the cells and associated with cell processes of osteogenic differentiated ASCs onto the DAM (Fig. 3c), and inside the DAM (Fig. 3d). EDS measurements performed on the mineral phase deposited by ASCs on DAM in the presence of commercial induction medium revealed an average Ca/P ratio of 1.52 (± 0.04).

**Fig. 4:**
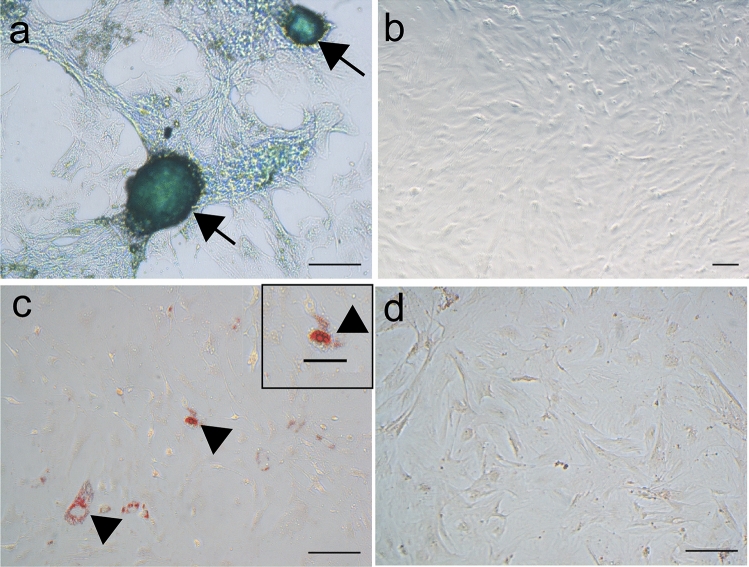
ASCs multilineage differentiation demonstrated by the presence of sulfated proteoglycans on ASCs micromass (**a**, arrow) and intracellular lipid-filled vacuoles (**b**, arrowhead) after culture with chondrogenic (**a**) and adipogenic induction media (**c**), respectively. ASCs maintained in regular medium were processed as negative controls, for chondrogenic (**b**) and adipogenic (**d**) differentiation. Alcian Blue stain (**a**, **b**); Oil-red stain (**c**, **d**); Objectives × 10 (**b**), × 20 (**a**, **c**, **d**); Scale bars 100 µm (**a**–**d**), 50 µm (inset in **c**)

Chondrogenic differentiation was observed three weeks post-induction with commercial chondrogenic differentiation medium (Fig. 4a), and adipogenic differentiation by two weeks with prepared adipogenic differentiation medium (Fig. 4c).

**Fig. 5:**
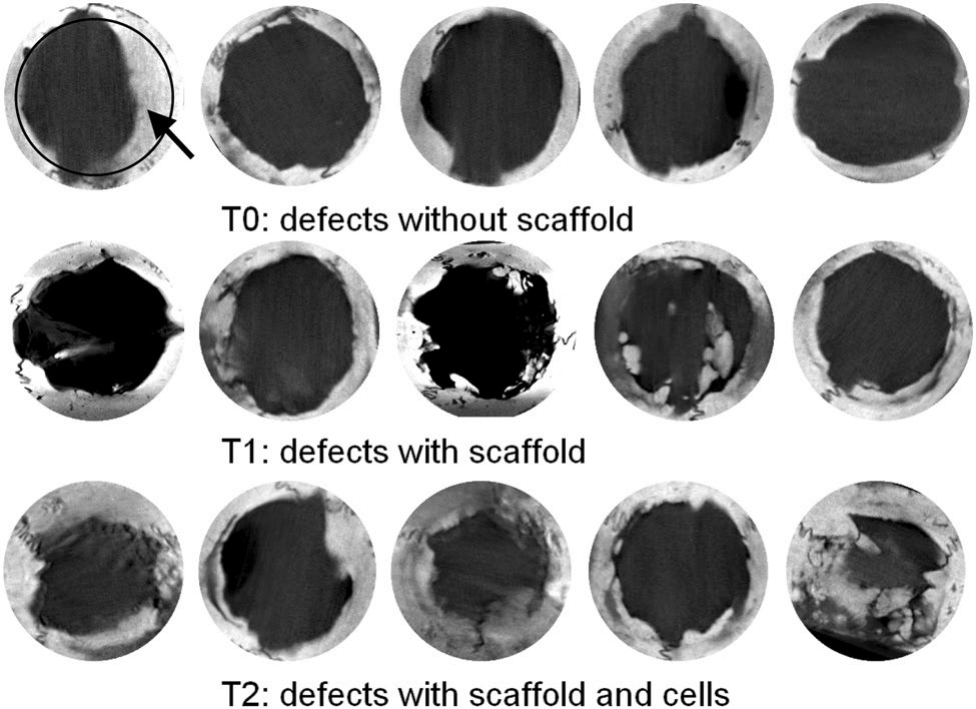
Micro-computed tomography (micro-CT) imaging of rat calvarial defects after 12 weeks of healing, with different treatments. Images of the defects, without scaffold (control, T0), treated with decellularized amniotic membranes (DAM, T1) and DAM with Adipose-derived stromal cells (DAM + ASCs, T2). Bone (arrow) deposited from the defect margins (circle in first picture) toward the center. The original defect size with 8 mm in diameter, circular images (10 mm in diameter)

Analysis of the micro-CT images revealed bone formation at the site of the defects in all three groups observed, from adjacent native bone. A centripetal in vivo ossification pattern was observed with bone deposition from and higher on the defect margins. Bone deposition closer to the defect central area was observed in defects with membrane or membrane associated with cells (Fig. 5), but without complete bony bridging.

**Fig. 6:**
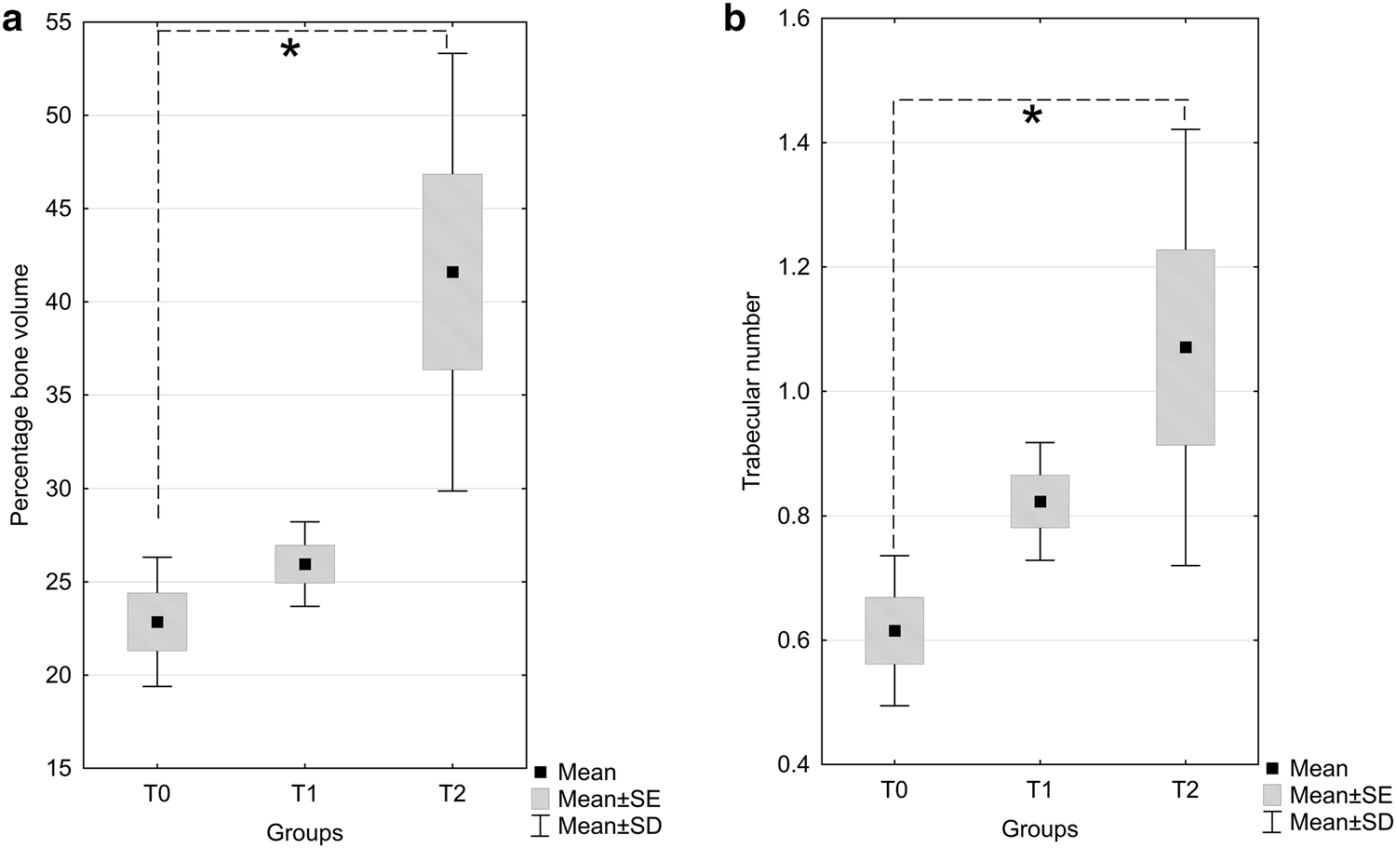
Box plot graphs of bone volume percentage (%, **a**) and trabecular number (1/mm, **b**) as detected by micro-computed tomography, 12 weeks after treatment of calvarial defects by implantation of decellularized amniotic membrane scaffold only (T1), decellularized amniotic membrane scaffold associated with adipose-derived stromal cells (T2), or no treatment (T0). Data represent means ± standard error (SE), ± standard deviation (SD); Bone volume percentage: Kruskal–Wallis test, Trabecular number: ANOVA/ Tukey’s test, *p < 0.05

The statistical analysis of calvarial bone healing parameters from micro-CT revealed significant difference on percentage of new bone volume to total bone (BV/TV), and trabecular number (1/mm). Kruskal–Wallis test was conducted to examine the differences on bone volume percentage according to the types of treatment. Significant differences were found among the three independent groups (Kruskal–Wallis-H (2,15) = 9.62, *p* = 0.0081). Pairwise test between groups showed that only the difference between T2 and T0 group was significant (Kolmogorov–Smirnov test *p* < 0.025). The significant increase in bone volume percentage was observed with the treatment with DAM associated with ASCs (T2 = 41.59 ± 5.24), compared to control (T0 = 22.85 ± 1.54), but not with DAM alone (T1 = 25.95 ± 1.01) (Fig. 6a). The higher repair observed in DAM grafted defects was evidenced after ANOVA/ Tukey’s tests (*F*(2,12) = 5.3228, *p* = 0.0221) by a significantly higher number of trabeculae (1/mm) in treatment with DAM associated with ASCs (T2 = 1.07 ± 0.15), compared to control T0 (0.61 ± 0.05), but without difference to implanted DAM in T1 (0.82 ± 0.04) (Fig. 6b).

**Fig. 7:**
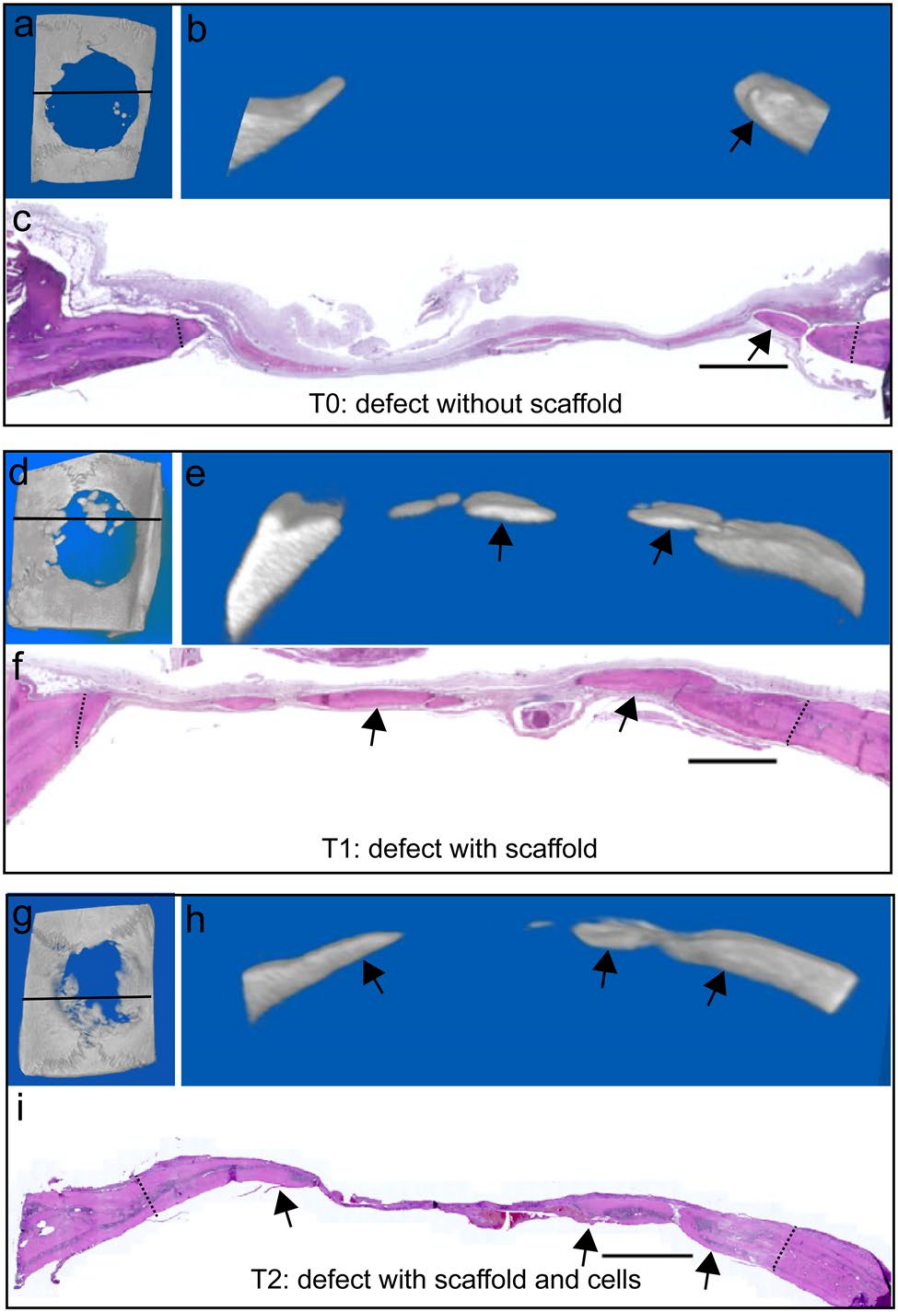
Bone repair of calvarial defects: without treatment (T0, **a**–**c**), treatment with DAM scaffold (T1, **d**–**f**), and with DAM associated with transplanted ASCs (T2, **g**–**i**). Correspondence of images from defect micro-CT of the complete defect (**a**, **d**, **g**) with frontal view position indicated by continuous line, frontal plane (**b**, **e**, **h**), and corresponding histological section (**c**, **f**, **i**) of the micro-CT, with the meningeal side toward the bottom of the section. Bone deposition (paired arrows) from original defect edges (dotted lines) following the periosteum and the collagenous scaffold. H&E stain; Scale bars 1000 µm

**Fig. 8:**
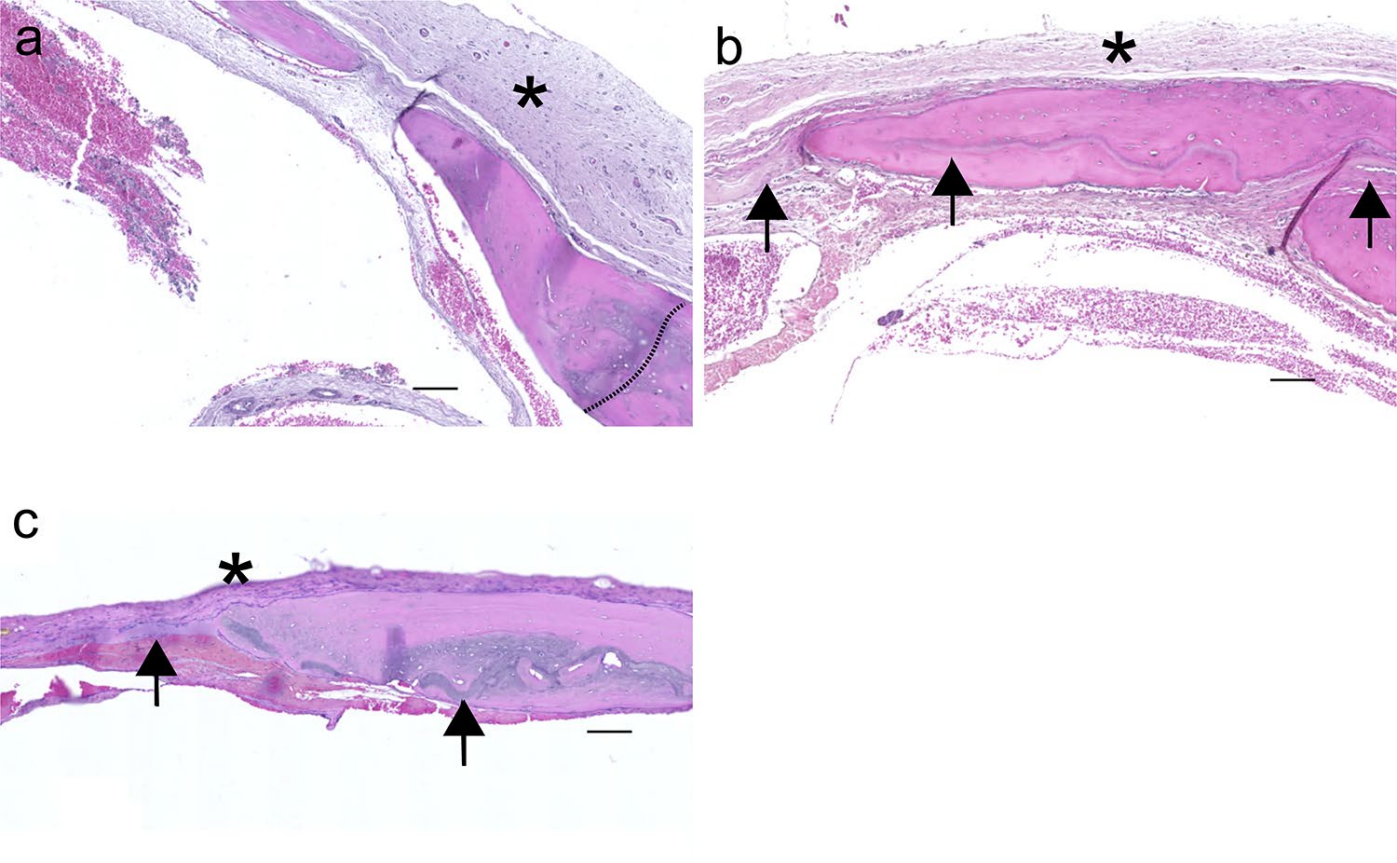
Detailed bone deposition from specimens without treatment (T0, **a**), treatment with DAM scaffold (T1, **b**), and with DAM associated with transplanted ASCs (T2, **c**). Bone deposited onto the defect margin (dotted line) associated with the inner cambium or osteogenic layer of the host periosteum, adjacent to its fibrous layer (asterisk) (**a**). Osteoconduction with DAM layers (arrow) observed adjacent to newly deposited bone, inserted into the bone matrix, and associated with the inner periosteum layer toward the center of the defect (asterisk) (**b**). Osteoconduction with DAM and ASCs, with DAM scaffold inserted into the bone (arrow) and associated with periosteum (asterisk) (**c**). H&E stain; Objective × 2.5; Scale bars 100 µm

**Fig. 9:**
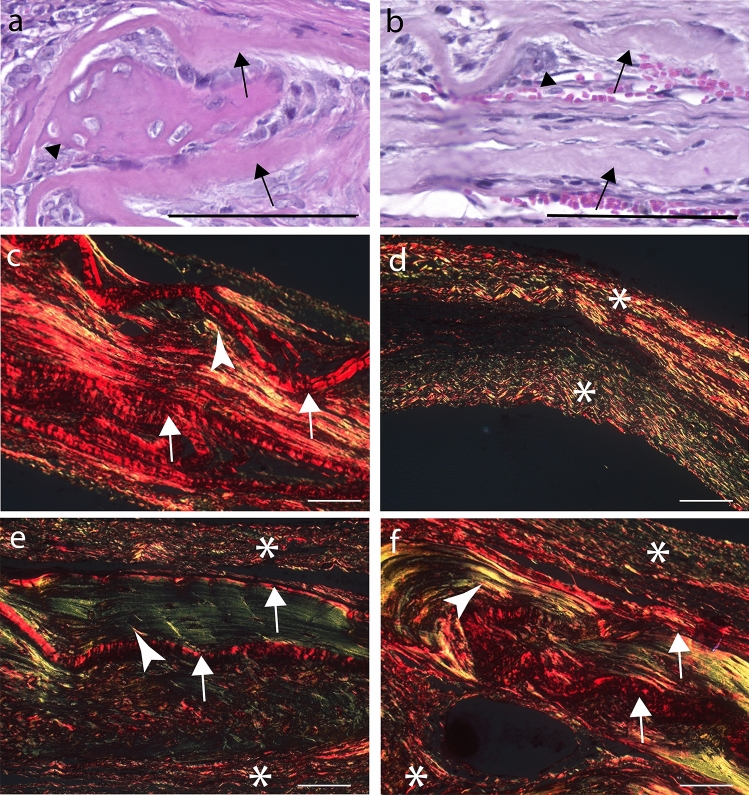
Details of DAM scaffolds in calvarial bone repair. Bone (arrowhead) deposited in the concave area between two transplanted DAM layers (arrow) (**a**). Osteoconduction established by vascularization between amniotic membrane layers (arrows), cell migration between collagenous fibers of the amniotic membrane, and creation of niches for bone deposition (arrowhead) (**b**). PRS-POL image of the specimen from **b** exhibits the DAM as bright red fiber agglomerate (arrow), interposed by bright yellow endogenous collagen (arrowhead) (**c**). PRS-POL images of specimens from three groups: T0 without treatment (**d**), T1 treatment with DAM scaffold (**e**), and T2 with DAM associated with transplanted ASCs (**f**), with the meningeal side toward the bottom of the section. Endogenous fibrous tissue from T0 (**d**), displaying upper and lower parts collapsed (asterisk), without matrix in the middle. Treatments with DAM (**e** T1; **f** T2) exhibit the transplanted collagenous DAM as bright red fiber agglomerate (arrow) interposed between upper and on lower fibrous tissue (asterisk), and bright green/yellow endogenous collagen and bone (arrowhead) deposited in the middle. H&E stain (**a**, **b**), PRS stain (**c**–**f**); Objective × 20 (**a**, **b**), Objective 10X (**c**–**f**); Scale Bars 100 µm

Histological analysis with H&E stain (Figs. 7–9) and PCR staining (Fig. 9) confirmed Micro-CT findings. Defects without treatment presented less bone deposited at the defect margins, and it merged with a thin fibrous host periosteum and the connective tissue layer covering the inner area of the defect, characteristic of defect healing with less bone tissue repair (Fig. 7a–c). The fibrous tissue covering the central part of the defect was thinner than the original well-vascularized bone tissue in the absence of membrane, with a connective tissue collapse observed in untreated defects (Fig. 7c). The effect of the DAM scaffold on osteoconduction was observed by providing anchorage for bone tissue deposited in the defects (Fig. 7d–i).

Bone deposited in calvarial defects treated with DAM, such as margin extensions or islets, had amniotic membrane-embedded or as a lining (Fig. 8). Newly formed bone from the margins of defects treated with membranes followed the scaffold orientation toward the center of the defect (Figs. 7, 8). Remnants of the decellularized amniotic membrane were observed in both membrane treatments as a homogeneous fibrous material, restricted into the defect, and maintained the integrity during the observation period without stimulation of inflammatory and immune response.

DAM scaffold enabled the ingrowth of vessels, stem cells, and osteoblasts from the host bone (Fig. 9). Collagen organization in the defect area was evaluated by PRS-POL, where collagen appears bright red, yellow, or green, in sharp contrast with the rest of the tissue that remains dark (Fig. 9). Collagenous niches for osteogenesis were observed on DAM concave areas from slightly undulated membrane scaffolds (Fig. 9) and vascularized connective tissue with endogenous cells propagated between the DAM layers or penetrated between the collagenous matrix, which provided expanded collagenous niches for osteoconduction and DAM incorporation into newly deposited bone (Fig. 9b, c). The amniotic membrane layers occupied the treated defect space (Fig. 9e, f), distinct from the defects without a collagenous matrix (Fig. 9d), regardless of whether they were associated with ASCs or not. Statistical analysis of the total fiber quantification from consecutive PSR-POL images of different treatments demonstrated a significant increase of fiber content (ANOVA/ Tukey’s tests: *F* (2,132) = 6.3873, *p* = 0.0022) between both DAM treatments (T2 = 30.73 ± 1.91; T1 = 34.21 ± 2.24) and control (T0 = 23.08 ± 2.55).

## Discussion

In the present work, the combined transplantation approach using ASCs and DAM, demonstrated enhanced calvarial bone regeneration. The decellularization process preserved the membranous structure integrity of the amniotic membrane, which was not fragile or difficult to handle. DAM provided a more biomimetic niche than the culture dish surface [20], biocompatible, and stable during the extended in vitro culture period for ASCs osteoblastic differentiation. Mineralized bone-like matrix deposited by ASCs was formed by calcium phosphate-containing globular accretions, as also demonstrate previously by detailed ultrastructural and mineral phase analyses [21]. The average Ca/*P* ratio of 1.52 (± 0.04) of the deposited bone-like matrix indicates a poorly crystalline hydroxyapatite, observed on other osteoinductive cell culture studies, also reflecting the variations in physiological bone development, and mineralization mechanisms [21].

Four DAM layers made it possible to cover the entire inner calvarial defect perimeter, increasing the scaffold thickness, and replicating the use of multilayered cell sheets [22]. A tridimensional cell sheet strategy with the layers of DAM associated with ASCs placed directly to the host site with minimal cell manipulation improved the bone regeneration potential with more vessel stability than single-cell sheet. The DAM scaffold provided an osteoconductive environment for cell migration and angiogenesis, and were incorporated concomitant with host bone deposition, without inducing an inflammatory and immune response, or presenting accelerated scaffold biodegradation. DAM was osseointegrated, with direct bone deposition onto the scaffold, creating a mineralized interface between DAM and newly deposited bone, or with the inclusion of the collagenous DAM as part of the newly deposited bone. There are controversial opinions regarding scaffold degradation, with fast degradation in vitro contraindicating their use for cell transplantation [23], because prolonged stability is necessary for cell support [24], versus rapid degradation as advantageous for cell release in the early period of osseous defect repair [25].

The amniotic membrane processing for cell removal and sterilization results in a matrix composed mainly of collagen types I and III, responsible for the membrane mechanical integrity, and types V and VI [26]. The bone matrix components are collagen fibrils, non-collagen interfibrillar proteins that make up the bone organic matrix, and the inorganic substance. Type I collagen is the major component of the organic matrix framework of bone, deposited by osteoblasts during bone formation, fracture repair, and bone resorption sites. Therefore, the presence of the DAM collagen favored the osteogenic process as a substrate for cell migration, attachment, proliferation, and matrix deposition.

The significant increase of collagen content provided by DAM treatments supported neovascularization from the defect to scaffold, surrounded by endogenous periosteal cells, promoting osteoconduction. Support for neovascularization was observed between the layers of DAMs and between loose collagenous fibers of the DAM.

DAM concave niches demonstrated the osteogenic potential of these areas, providing sites for initial islet bone deposition, with subsequent incorporation into the lamellar bone as undulated DAMs inside newly deposited bone. Previous reports documented those scaffold concavities are conducive for osteogenesis [27]. Quantification of total collagen, from DAM scaffold and endogenous origin, and characterization of the collagen structure was done after PSR-POL, without distinguishing their type or origin, since the orientation, thickness, and packing of collagen bundles determine the amount of polarized light absorbed by the stain.

Stem cell participation in bone tissue regeneration may depend on cell attachment and proliferation on scaffolds, subsequent differentiation, and integration into the surrounding tissues [6]. In vitro results demonstrated ASCs' attachment, cell proliferation, and osteogenic differentiation on DAM. One limitation of the present study was the lack of a cell marker for tracking transplanted cell fate and identifying cell participation in the healing process. Even though the contribution of the ASCs for bone healing was not established, this data demonstrated higher repair of critical-size bone defects with transplanted cells and emphasized the indication of the scaffolds prepared with DAM and ASCs in bone tissue engineering. Transplanted ASCs in bone sites may be differentiated into osteoblasts or stimulate endogenous healing by trophic function [28]. New bone in calvarial defects were determined to consist mainly of transplanted cells [29], and ASCs directly differentiated into osteogenic cells in vivo [30]. Additional improvements in bone tissue engineering have been reported, with complete bony bridging in calvarial healing, by preserving cell stemness during long-term in vitro expansion [22], and after ex vivo osteogenic priming of mesenchymal cells [31].

Osteogenesis through the osteoconduction process, providing a structural scaffold to support vascularization and host cell reconstruction, can explain the successful bone augmentation observed on both DAM treatments. Furthermore, the association with ASCs may have allowed osteogenesis through site osteoinduction, observed by the significantly higher bone deposition with DAM and ASCs grafts. The role of the anatomical defect region was determinant for the results obtained in the present study. The calvarial bone defect model, as an orthotopic non-load-bearing site, provided an appropriate osteogenic environment to assess the effect of non-induced ASCs for bone tissue engineering [29]. Accelerated bone healing of calvarial defect has been reported in other studies with ASCs, associated with commercial bone graft in rabbit [32], and with PLGA/HA [29, 31]. Investigators also observed significant healing improvement with ASCs without previous cell induction, genetic manipulation, or association with exogenous growth factors [28, 29, 33–36]. Untreated ASCs as control also stimulated healing [28, 37–39]. Osteoinduction of ASCs has been investigated in calvarial bone healing models [40, 41], besides cell transfection with vascular endothelial growth factor (VEGF) gene sequence [38], and cell transduction for sustained expression of BMP and Stromal cell-derived factor [42].

The pro-osteogenic paracrine effect of ASCs was identified between transplanted ASCs and host osteoblasts, through BMP and Hedgehog signaling [14], stimulating the endogenous healing response. ASCs may also accelerate new bone formation by promoting angiogenesis through pro-angiogenic paracrine factors [40, 43–45], and secretion of VEGF [44, 46]. ASCs may participate directly in vasculogenesis, stabilizing endothelial networks by developing pericyte characteristics [43]. Strategies to stimulate bone regeneration are the selection of perivascular cell subsets from SVF [47, 48], and ASCs–derived exosomes [49]. The extended clinical therapeutic use of ASCs for bone regeneration still requires a standard for cell characterization and culture.

## Conclusion

DAM confirmed potential and efficacy for ASCs transplantation in this bone engineering model. In vivo DAM treatments demonstrated osteoconduction, providing a collagenous structural matrix for endogenous cell migration, neovascularization, bone tissue deposition, and anchorage. Bone deposited between the DAM layers, especially on niches formed by membrane undulation, or between the collagenous fibers of the DAM, incorporating the DAM into the newly deposited tissue.

The further association of DAM with ASCs, stimulated the healing of critical-sized adult rat calvarial defects, demonstrated by DAM graft incorporation concomitant with higher host bone deposition, compared with defects without any treatment. This association of the ASCs and DAM offers advantages for optimizing bone regeneration in this model.

Future research will be necessary with the association of bioactive materials and in different defect models since DAM with ASCs may also be used for supporting vascularization, periosteal cell migration, and bone healing with implants, even in load-bearing defects.
